# Complete genome sequence of pathogenic *Pseudomonas aeruginosa* strain P4 associated with persistent lung infections

**DOI:** 10.1128/mra.00488-25

**Published:** 2025-10-24

**Authors:** Ravali Arugonda, Namrata Bonde, Nicholas Dillon

**Affiliations:** 1 Department of Biological Sciences, The University of Texas at Dallas 12335, Richardson, Texas, USA; Loyola University Chicago, Chicago, Illinois, USA

**Keywords:** *Pseudomonas aeruginosa*, pathogen, genomes

## Abstract

Here we are reporting the circularized whole genome sequence of multi-drug resistant *Pseudomonas aeruginosa* strain P4, a clinical human lung isolate. This strain has previously been used to study antibiotic resistance in *P. aeruginosa*.

## ANNOUNCEMENT

Multi-drug resistant (MDR) *Pseudomonas aeruginosa* is one of the ESKAPE pathogens and a leading cause of morbidity and mortality (1). In this announcement, we report the assembled complete genome sequence of *P. aeruginosa* strain P4, a clinical sputum sample originally obtained from a tertiary academic hospital in the New York metropolitan area (2). The strain was kindly provided to us by Dr. Victor Nizet (UCSD). Previous studies have been done on P4 in evaluating synergistic antibiotic combinations and antimicrobial peptides (2–4), understanding the effect of host environmental factors on immune responses (5), and in non-antibiotic disinfection strategies (6, 7). Here, we present a complete, circularized genome assembly of P4, comprising a chromosome and one plasmid. This published genome will be useful for studying the metabolism, mechanisms of antibiotic resistance, and virulence of the clinical *P. aeruginosa* strain P4. Genome assembly and annotation metrics are summarized in Table 1.

**TABLE 1:** Genomic characteristics of *P. aeruginosa* strain P4, including circularized chromosome and plasmid^*a*^

Genomic features (P4)	Chromosome	Plasmid
Strain ID	P4	
SRA accession no.	SRR33560916 SRR33560917	
GenBank accession no.	CP187226	CP187227
Total no. of raw nanopore reads	239,640	
N_50_ of nanopore reads (bp)	11,394	
Total no. of Illumina reads (R1 +R2)	7,369,882	
No. of contigs	1	
Total length (bp)	6,670,990	47,865
Coverage of total genome (×)	319	
GC content (%)	66.25	59.12
No. of predicted genes	6,339	

^*a*^ Genomic statistics are based on the hybrid assembly of Nanopore and Illumina sequencing data.

DNA from P4 was extracted following the Qiagen DNeasy Blood and Tissue kit (Cat#69504) instructions. Adapted from (8), P4 DNA was isolated via first resuspension of pellets from overnight cultures grown in cation-adjusted Mueller Hinton Broth (CA-MHB) at 37°C, 200 rpm in 1× TAE buffer (Tris, Acetic acid, EDTA) followed by centrifugation and resuspension in enzymatic lysis buffer (20 mM Tris-Cl, 2 mM EDTA, 1.2% Triton-X-100) and 50 mg/mL lysozyme. RNase A was then added, and the mixture was incubated at 37°C for 2 h. 25 µL of Proteinase K and 200 µL of AL buffer were added and incubated for 30 min at 56°C on a heat block (VWR). Qiagen kit instructions were followed exactly for the ethanol addition and DNA elution.

Hybrid sequencing was performed by SeqCenter (Pittsburgh, PA). Illumina sequencing libraries were prepared using the tagmentation-based and PCR-based Illumina DNA Prep kit and custom IDT 10 bp unique dual indices with a target insert size of 280 bp. Illumina sequencing was performed on an Illumina NovaSeq 6000 sequencer, producing 2×151 bp paired-end reads. Demultiplexing, quality control, and adapter trimming were performed with bcl-convert (v4.2.4).

Sample libraries were prepared using the PCR-free Oxford Nanopore Technologies (ONT) Ligation Sequencing Kit (SQK-NBD114.24) with the NEBNext Companion Module (E7180L), following the manufacturer’s instructions. No additional DNA fragmentation or size selection was performed. Sequencing was carried out on an ONT GridION platform using R10.4.1 flow cells in multiplexed runs, with the 400 bp sequencing mode and a minimum read length threshold of 200 bp. Adaptive sampling was not enabled. Guppy (v6.5.7) was used for super-accurate basecalling, demultiplexing, and adapter trimming.

Raw sequenced reads were quality controlled using FastQC (v0.12.1) (9). Trimmomatic (v0.39) (10) trimmed low-quality sequences from raw Illumina reads. Nanofilt (v2.8.0) (11) and porechop (v0.2.4) (12) were used to trim the residual adapter sequence from ONT reads. Illumina and ONT reads were assembled using Unicycler (v0.4.8) (13). Once the assembly was done, contigs were checked for circularization using circlator (v1.5.5) (14). Genome annotation was done using the NCBI Prokaryotic Genome Annotation Pipeline (v6.6) (15). The default parameters were used for all software.

## Data Availability

The complete circularized genome sequence of *P. aeruginosa* strain P4 has been deposited in GenBank under accession numbers CP187226 (chromosome) and CP187227 (plasmid). Raw sequencing reads are available in the Sequence Read Archive (SRA) under accession numbers SRR33560916 (Illumina) and SRR33560917 (Nanopore).
